# M.M. Andral and Lerminier on Diseases of the Abdominal Viscera, from the Clinique of La Charité, &c.

**Published:** 1827-07

**Authors:** 


*Clinique Medicale, ou Choix d'Observations, Recueillies à la Clinique de M. Lerminier, Medecin de l'Hôpital de* LA CHARITE, *&c. et Publiés sous ses yieux.* Par G. ANDRAL, fils. Tome Quatrieme. Maladies de l'Abdomen. 8vo. pp, 668. Paris, 1827. Balliere, Bedford-street, London.

### ABDOMINAL DISEASES.

IN the Periscope of our Eleventh Number, (January, 1827) we gave a very extended account of M. Andral's Memoir on the Morbid Alterations of the Mucous Membrane of the Stomach, condensed from the REPERTOIRE, in which the memoir originally appeared. This memoir forms the first chapter of the division of M. Andral's volume now under consideration, and shall therefore be passed over. We now proceed to the other chapters in succession.

### CHAP. II.

"*Are there any special symptoms by which we can distinguish scirrhus and cancer of the stomach from other lesions of that organ?*"

These symptoms are of three kinds—the first are purely local, consisting of disturbances in the functions of the stomach—the second relate to the function of general nutrition, which is necessarily disturbed by gastric disorders—the third set of symptoms are purely sympathetic. Here it may be asked, are the different alterations of the mucous membrane, described in the first chapter,* to be recognized by any special symptoms during life? M. Andral answers in the negative. With the exception of some symptoms peculiar to mechanical obstruction of the cardiac or pyloric orifices of the stomach, by tumours, the same functional derangements will often be developed by the various organic lesions there described. Thus, it is a great mistake to suppose that lancinating pains are peculiar to that disease which we denominate cancer or scirrhus of the stomach. On the contrary, M. Andral undertakes to prove that these lancinating pains are comparatively rare accompaniments of this terrible disease—indeed, he is not quite sure that he ever saw any unequivocal instance of this accompaniment. Among the numerous individuals whom he and M. Lerminier have examined after death, and who presented scirrhous and cancerous affections of this organ, many never complained of any pain in the epigastrium—others complained of only a sense of constriction or weight there—some complained of tenderness on pressure—while many others could bear pressure without any inconvenience. In a considerable proportion, pain was only complained of after taking food. In short, on comparing the symptoms of those who merely laboured under gastric irritation or

* See Med. Chir. Rev. for January, 1827, from page 145 to page 161.

chronic phlogosis, with those in whom there was actual scirrhus or cancer, there were none which could be considered as characteristic of the latter or graver malady.

If we look for more certain diagnostic marks in the various derangements of the digestive function, we shall be equally disappointed: thus, in taking two extreme cases as examples, they have seen individuals who, during life, exhibited no other morbid phenomena than anorexia, more or less constriction at the epigastrium, and some uneasiness after eating—and yet, after death, they found vast cancerous ulcerations, or extensive scirrhous indurations of the stomach. On the other hand, they have seen individuals who experienced great pain and misery after eating, with vomitings, acrid eructations, &c. and yet, on dissection, nothing amiss could be discovered in the stomach, except perhaps a little redness or softness of the mucous membrane.*

Will the qualities of the matters cast up by vomiting throw any light on the subject? It is said by authors that hæmatemesis is exclusively connected with the existence of fungous vegetations, cancerous ulcers, or softened encephaloid masses in the stomach. They have said that these diseases produced discharges like suet, or coffee grounds: and no doubt this is often the case; but the same kind of discharges not unfrequently occur under very different gastric affections, as mere injection of the vessels of the mucous membrane, or some slight thickening of this coat.

As to the general symptoms, whether sympathetic derangements, or defects of chylification, they are not better calculated than the local symptoms, to discriminate with certainty between the different organic lesions. At the same time it is certain that the pale yellow tint of the countenance, the emaciation, and the prostration of strength, are very characteristic of scirrhous or cancerous disease of the main organ of digestion.†

* What will Dr. Philip say to this, who so confidently discriminates between the different *stages* of indigestion—between irritation and inflammation, merely by certain feelings communicated to the finger by the pulse—or by tenderness on pressure of the epigastrium!

† We lately attended a patient, (with Mr Dickenson a very intelligent surgeon, of Sloane Street,) who, for about five months, had been troubled with gastric disorder, which had progressively increased. Slight uneasiness a few hours after eating, was the first symptom—then sickness and vomiting, at longer or shorter intervals, with emaciation. Two months before death, when we first saw the patient, the vomiting occurred every day, or every second day, four, six, or ten hours after the principal meal—the matters thrown up being chiefly the remains of half digested food. He had little or no pain, except after eating. Upon careful examination, we thought we could perceive some little induration in the region of the pylorus, and leechings, counter-irritation, and farinaceous food were tried but with little success. He got impatient, and resorted to several other medical men, and Mr. Earle among the rest. This gentleman ordered him to live solely on milk, taken in small quantities at a time. The effect was surprising. He lost his sickness and uneasiness—and returned to his avocation, (that of

From what has preceded, we may come to the conclusion that, unless a tumour can be actually felt in the region of the stomach, there is no diagnostic symptom by which we can distinguish what is called scirrhus or cancer of the stomach from chronic inflammation or irritation of that organ.

### Chap. III.

### *On Derangements of Function in the Stomach without any appreciable Lesion of Structure, in Individuals who had been affected with Chronic Gastritis.*

Among those individuals whom our authors (for we speak of M. M. Lerminier and Andral) have seen perish under the various shades of chronic gastritis, some have sunk gradually, without the appearance of any other disease, but merely from debility, often without any febrile movement in the system till the last. In other cases the chronic passes into the acute form of gastritis, when fever, of a low type, then generally supervenes, in which the patient succumbs. In a good number of cases, however, death is occasioned by the supervention of a disease in some other part, as acute inflammation in the intestinal canal, in the peritoneum—perforation of the stomach itself, &c. In some few instances, our authors have seen patients affected with chronic gastritis die of cerebral disorder, that had supervened on the original malady. In such cases, the most careful dissection could not detect any appreciable lesion, either in the stomach or brain. The following cases are interesting, under this point of view.

*Case* 1. A cook, aged 38 years, had laboured under symptoms of indigestion for a long time. He vomited up the remains of his food, every two or three days; but affirmed that he never felt pain in the epigastrium. The tongue was white, the emaciation considerable,

---

master tailor,) concluding that he had, at last, found out a medical man who perfectly understood his complaint—not without some strong suspicions that all the others were mere bunglers. For a whole week, or more, he never vomited; but the fairy prospect of recovery then vanished—for ever! The gastric irritability returned worse than before—he emaciated rapidly—and died the most complete skeleton we ever witnessed. We assisted Mr. Dickenson at the *post mortem* examination, in presence of Mr. James Boyle and others. The Stomach was of extraordinary dimensions. The pyloric orifice was almost obliterated. It would not admit the point of the little finger. This orifice was surrounded with a scirrhous induration nearly as hard as gristle, which gradually lost itself in the coats of the stomach. There was no breach of structure in the mucous membrane; but about an inch and a half from the pylorus, there were the unequivocal remains of a former ulcer completely healed, the size of a shilling, and depressed, at least a line beneath the level of the surrounding parts. Upon examining his wife, we learnt that six or seven years previous to his death, he had laboured under a severe stomach complaint, for several months, but from which he had entirely recovered.

pulse rather accelerated, temperature of skin natural. Three weeks passed without any change in these symptoms, when, on the 11th June, there appeared to be some wandering of the ideas. He kept his eyes fixed on the ceiling of the room. Blisters to the legs. In the course of the night the mind became more deranged, and on the 12th there was complete delirium. The patient lay on his back, his eyes turned up, and his mind apparently in a state of total abstraction. He would not reply to any questions, but only pronounced, from time to time, some unintelligible words. The pulse was quick, the skin moderately hot. 13th, The left pupil was strongly contracted, and the conjunctiva of the eye much injected. He appeared blind of this eye. The sense of hearing also appeared very obtuse—pulse extremely quick, but the skin not hot. He died the next day, without any particular symptom beyond what has been described.

*Dissection.* The meninges of the brain were free from injection, white, and transparent. The lateral ventricles contained a very small quantity of limpid serum, and the same was found, in very moderate proportion at the base of the skull. The cerebral substance was generally pale, and appeared sound in all respects. The thoracic organs were in a state of integrity. In the neighbourhood of the pylorus, the coats of the stomach were considerably thickened, and rather homogeneous in consistence. In the great arch of the stomach, there was an abrasion of the mucous membrane, the size of a thirty-sous piece. The bottom of this ulcer was formed of thickened cellular membrane. In every other part, the stomach was sound. There were some points of injection in the mucous lining of the intestines ; but generally speaking, they were very pale. No other disease in the abdominal viscera.

Our authors ask if the cerebral symptoms which appeared, and which indeed caused the death of the patient, could be called meningitis, or merely a sympathetic affection of the cerebral functions, independent of phlogosis? We think the last conclusion must be come to, even by the stanchest supporters of the doctrine of Clutterbuck. The above case shews that irritation of the brain and nervous system may supervene on stomach-disease, without any transition of the chronic gastritis into the acute form.

*Case* 2. A woman, aged 40 years, experienced, for a long time, those symptoms which usually indicate organic disease of the stomach; as anorexia, tenderness or pain in the epigastrium ; sense of uneasiness and plenitude in the stomach, after taking food ; eructations ; vomitings, though rarely, and only when the mind was irritated by any moral cause ; constipation ; apyrexia. The patient was still rather embonpoint. About ten days after she came into the Charité, she was seized, without any known cause, with an attack of epilepsy. To these convulsions there succeeded a state of coma, with paralysis of the limbs, and rattling noise in the throat. She died 25 hours after this seizure.

*Dissection.* The pia mater covering the convexities of the cerebral hemispheres was injected and red, this redness appearing in stripes or patches. In no place was it so intense, however, as to render the membrane opaque. In other respects, the meninges appeared sound. The brain itself, and the spinal marrow were found without any appreciable lesion, their vessels being very moderately injected.

The stomach was greatly contracted, being not larger than a portion of intestine. The mucous membrane was of a slate colour, and thrown into numerous folds or wrinkles which, on accurate examination, appeared to be unequal thickenings of the membrane, and not produced by mere contraction of the muscular coat of this organ. There was nothing amiss in any other part of the body.

Here there was no organic lesion which could account for the epileptic seizure that caused the death of the patient. But it is not in cases of gastric affection alone that our authors have seen fatal cerebral disorder supervene. They have observed similar accidents, where the stomach was sound, and only some chronic inflammation in the intestines. The following is an example.

*Case* 3. Catherine, aged 38 years, a cook, and whose mother was insane, had always been subject to violent head-aches, which she attributed to determinations of blood to the head. She had not menstruated for ten years past. She entered LA CHARITÉ on the 29th September, 1826, and on the 30th presented the following symptoms:—eyes sparkling—pupils dilated, and but little sensible to light—no fixed pain in any part of the head—violent delirium, with intervals of sense, when she answered questions rationally—hallucinations of sight and hearing—pulse full, but easily compressed—respiration natural. By auscultation and percussion, the thoracic organs were found in a state of integrity. The tongue was dry, and slightly red, the papillæ salient—abdomen tender on pressure, especially in the epigastric region—constipation—urine scanty—the bladder appeared distended. Twenty-four leeches had been applied to the epigastrium; and 30 more were now ordered to the anus and vicinity—with sinapisms to the feet—lavements, &c.

*1st October;* eyes haggard and watery—lies in a state of tranquillity and obstinate taciturnity—jaws firmly locked—pulse small and frequent—slight heat of skin. The history is not continued; but on the 28th of October, it was decided to send the patient to the SALPETRIERE, as she was pronounced to be insane. She was accordingly sent to that establishment, but died suddenly the very next day, at two o'clock in the morning.

*Dissection.* The membranes of the brain and spinal marrow, examined with the greatest care, presented no appreciable change of structure. The same might be said of the cerebral and spinal brain themselves. There was a small quantity of limpid serum in the lateral ventricles. Nothing particular in the thoracic viscera. A portion of the mucous membrane of the stomach, the size of a crown piece, situated in the great arch, was injected, and of a dark colour. The mucous membrane of the cæcum was also injected. Throughout the whole of the colon the mucous follicles were strongly developed, and chronically inflamed. The mucous membrane, in the interstices, was not altered. The colon represented a triangle, the apex of which corresponded with the xiphoid cartilage, and the base with the hypogastric region. The uterus and its appendices were in a natural state.

Here there was no structural lesion in the brain to account for the death of the patient, and the only disease, of any standing, was the chronic follicular inflammation in the colon.

We now come to the second division of this chapter, in which the authors treat of nervous accidents resulting from acute inflammation of the mucous membrane of the stomach. Some of these cases we shall touch upon in this place.

*Case* 4. A man of middle age, was seized, without apparent cause, (four days prior to his entrance into the hospital) with abundant bilious vomitings, pain in the epigastrium, and fever. In about twenty-five hours after the commencement of these symptoms the patient began to experience a difficulty in moving the lower jaw, which soon amounted to violent trismus, that continued two days. He was then conveyed to LA CHARITÉ, where he presented the following phenomena, viz.:—trismus—stiffness of the upper and lower extremities—abdominal muscles as hard as a board—intellectual functions undisturbed. He was able to relate his own case, notwithstanding the trismus—had no pain in the epigastrium—no vomiting. He was carried into one of the surgical wards, and died the same evening.

*Dissection.* Very inconsiderable injection of the meninges—slight red points in the cerebral substance—no appreciable alteration of structure in any part of the encephalic mass, on the most careful examination. The spinal marrow and its envelopes were perfectly healthy. The mucous membrane of the stomach was of an intensely red colour throughout its whole extent, which red colour could not be seen till a stratum of thick mucus was removed. The colour was found to be caused by very minute injection of the capillary vessels. The mucous membrane was not softened. The rest of the digestive canal was healthy, as was every other viscus in the abdomen and thorax.

The authors remark that they have rarely witnessed such an intensely red colour of the stomach, or to such an extent, as in the present instance. The gastric affection was well marked in the beginning; but no sooner did the tetanic symptoms shew themselves, than those of the gastric inflammation became obscure. The transmission of the sympathetic irritation from the stomach to the spinal nervous system can only be accounted for by some predisposition in the latter organ, anterior to the gastric inflammation. The authors saw a case in LA CHARITÉ, where a man was affected with chronic pleurisy, for which a seton was inserted in the side. Immediately after this insertion, the man was seized with tetanus, of which he died.

*Case* 5. A wine dealer, aged 33 years, felt, on the 8th of October, a sense of weight in his head, more uncomfortable than painful. In the evening, there was general malaise—delirium in the course of the night. He was received into La Charité on the 9th. He complained to the house-surgeon of giddiness, tinnitus aurium, and he had some diarrhœa. In the night delirium returned. On the 10th, at the morning visit, he presented the following symptoms:—remarkable exaltation of ideas—great loquacity, yet with the power of answering pretty distinctly—extreme quickness in all his motions—face flushed—expression of the eyes natural—pulse full, hard, moderately quick—temperature of the skin natural—tongue clean and moist—abdomen soft, and bowels rather loose. As the brain seemed to be the seat of a sanguineous congestion, he was bled from the arm to 12 ounces, and diluents were exhibited. No alteration during the day. He had two or three motions in the course of the evening and night. The pulse became accelerated, and delirium returned in the night. 11th, He was quite sensible this morning, and in the same state as yesterday. Bled again from the arm, and sixteen leeches applied to the neck. The blood, both times, was covered with a thick buff. In the evening the delirium returned as before, and in the course of the night he became furious, requiring four men to hold him. 12th, The delirium continues this morning—the face is greatly flushed, eyes sparkling, and rolling violently in their orbits, constant vociferation, streaming perspiration from the head and face, the rest of the surface being dry. The violent contortions of the arms prevented the physicians from ascertaining the state of the pulse. The tongue appeared natural. Thirty leeches applied to the neck—sixteen ounces of blood from the arm, with great difficulty. No amelioration of the symptoms followed this depletion, although the leech-bites bled freely. At half-past nine (an hour and a half after the bleeding) he ceased suddenly to vociferate—his face became tumid and red—his arms rigid—the breathing interrupted—and in five minutes more he expired.

*Dissection.* The arachnoid was transparent—the pia mater very slightly injected—a very little limpid serum in the lateral ventricles, not more than is generally found in all bodies—substance of the brain natural, but presenting numerous red points when sliced—lungs sound—left ventricle of the heart remarkably enlarged, without thickening of its parietes—internal surface of the stomach pale, as well as two-thirds of the small intestines. In the inferior third there existed numerous red stripes or patches of the mucous membrane, which, however, was neither more dense, soft, or thick than in the interstices of these patches. These stripes represented a number of circumscribed phlogoses, none of them having passed the first degree of inflammation, namely, injection of the capillaries and being without thickening or softening of the mucous membrane itself. The large intestines, and all the other abdominal viscera were perfectly sound.

It is observed by the authors that, in this individual, the cerebral symptoms were so strongly marked as to authorize the opinion that the membranes of the brain were affected with idiopathic inflammation. Yet the said membranes presented no trace of phlogosis, and it was only in a portion of the mucous membrane of the small intestines that any mark of inflammation could be found, on the most careful dissection. This last disease produced no evidence of its existence during life, unless it was the slight diarrhœa which has been recorded above. "Was the terrible irritation in the brain and nervous system then, derived sympathetically from the intestinal inflammation?" The solution of this question the authors leave to their readers, contenting themselves with faithfully recording the facts. The suddenness of death, without any intermediate state between violent action and extinction of life, may well excite the speculations of the physiologist and pathologist!

### CHAP. IV.

*Investigation of certain Morbid Conditions of the Stomach and Intestinal Canal, which may be advantageously treated by other means than Antiphlogistics.*

This is an important subject of examination, and imperiously calls for attention in these days, when all gastric affections, on both sides of the channel, are attacked by leeches. Hitherto our authors have brought forward diseases and disorders of the stomach, which, they think, demanded unequivocally the antiphlogistic treatment. "One of the greatest services," say they, "which have been rendered to humanity by M. Broussais, is that of having demonstrated that a group of symptoms generally attributed to debility of the stomach, were, on the contrary, connected with a state of *excitation* of that organ, requiring a soothing mode of treatment." This is very true. Bitters, tonics, and stimulants have been injudiciously exhibited by the routinists of France and England, where inappetency, emaciation, debility, and a thousand wretched feelings of mind and body were dependent on a morbid irritability of the gastric and intestinal nerves, which demanded reduction of diet, and the most unirritating medicines.

There is, however, observe our authors, another class of gastric affections, where the symptoms closely resemble those dependent on irritation or chronic inflammation of the mucous membrane, and yet where *post-mortem* examination can discover no appreciable lesion of that membrane. In such cases, the antiphlogistic treatment does not succeed, but rather aggravates the disorder.

"These cases are comparatively rare, but they are real. Thus we have seen many individuals in LA CHARITÉ who, during their sojourn in the hospital, had presented complete loss of appetite, without any nausea, vomiting, thirst, or pain in the epigastrium. They died of other chronic diseases, as of the lungs, lower portion of intestine, or liver; and, on dissection, there could be found no appreciable alteration of the stomach. In other instances, the individuals, besides complete anorexy, complained of a sense of weight and tightness in the epigastric region, either constantly, or after the ingestion of food. In others, still, there was actual rejection of the food taken, and yet after death no traces of disease in the stomach. These facts prove that the functions of the organ may be disturbed without any appreciable lesion of structure." "Who," say the authors, "can affirm that, in such cases, the functional disturbance is the result of irritation ?"

There certainly may be other disorders of the digestive function than those which arise from irritation or inflammation ; but from all that we have ever "seen, felt, heard, or understood," there is pretty generally a *morbid irritability* of the organ co-existing with these disordered conditions, from whatever cause they may spring. Our authors justly observe that the stomach sympathises so universally with other organs and structures of the body, that any disorder of these organs will necessarily disturb the function of digestion, without any necessity for the existence of either inflammation or irritation. Why should it not be with the internal as with the external surface of the body ? We see how chronic diseases modify the cutaneous envelope, in respect to its function, follicular and epidermic ; without any appearance of inflammation or irritation. Why, in similar chronic diseases, should we not have the functions of the stomach profoundly altered ? It seems indeed an universal, and, no doubt, wise law of the animal economy that, when other organs, as the lungs, liver, intestines, &c. are disturbed in their functions, the chymifactive process of the stomach shall be proportionately suspended or diminished ; for, of what use would be the formation of chyme in the stomach, if the other processes, by which the chyme is rendered fit for assimilation with the general fabric of the body, are imperfect ? The chymifactive process would then, in fact, if undiminished, aggravate the malady, instead of nourishing the body.

There are sometimes seen, in acute diseases, such disorder of the stomach, during life, as would lead one to expect alteration of structure after death. The following case is an example.

A child, three years of age, was seized with violent vomiting, without any known cause, which vomiting continued undiminished for 24 hours, without any other attendant symptom of consequence. The little patient then fell into a state of coma, which became more and more profound, till death ensued. The pulse had always continued very quick, but the tongue appeared natural. On dissection (in presence of the elder Andral and Dr. Descieux) the ventricles of the brain were found greatly distended with limpid serum, but no other morbid appearance in the brain or its appendages. The stomach was perfectly free from every appearance of disease, as were all the other organs of the body.

Here the violent gastric disorder was evidently produced sympathetically by the cerebral affection. It was one of the most marked cases of acute hydrocephalus, uncomplicated with any other disease, which our author has ever met with in practice.

From these and many other facts, it appears evident that the function of the stomach may be greatly deranged, without any appreciable alteration of its texture. And the same remark applies to other organs in the body, besides the stomach. We see diarrhœa and other instances of flux from the bowels, without change of structure—cases of dropsy without manifest obstruction to the venous circulation, without any antecedent inflammation, and without any cognizable modification of the serous membranes—finally, do we not see the nervous system constantly deranged in its functions, without our being able to recognize any alteration in its structure?

M. Andral, although he has laboured in a former paper (see No. xi. p. 160) to shew that many cases of softening of the mucous membrane of the stomach are connected with or dependent on some kind of chronic inflammation, yet acknowledges that there are many other cases, where chronic phlogosis cannot be taken into the account. "It appears to us that this softening which, in many people exhausted by chronic diseases, shews itself in the mucous membrane of the stomach, is only a part of that diminution of consistence which is seen in the muscular fibre, and even in the blood drawn from the veins of such patients." Indeed, it is by no means unreasonable to suppose that, in cases where the principal agents of life, the blood and the nervous system, neither nourish nor excite, in a proper manner, the organs of the body, the vital force of aggregation should also suffer in proportion, and fall below its normal physiological degree of intensity. Hence the diminution of cohesion in certain tissues, varying from what is vulgarly called "flaccidity of muscle," down to that condition where the solids of the body become semi-fluid. Thus the transparent cornea softens and becomes perforated where animals are insufficiently fed. M. Andral has seen the same thing in the human subject, both old and young. He has seen the central parts of the brain become almost fluid in children who had been miserably nourished, and who were in the last stage of marasmus. Yet this liquefaction of the cerebral substance was not denoted, during life, by any cognizable symptom of encephalic irritation or inflammation. Would it be a departure from sound physiology, says M. Andral, to place these softenings (ramollissemens) to the account of defect of nutrition or diminution of vital force? Who can affirm that all softenings of the structure of the heart are owing to chronic inflammation or irritation of that organ? The same may be observed of certain degenerations of the liver and spleen, of which, more has been said in another part of this volume. Finally, can the softenings of the bones, in ricketty children, be considered as a process of inflammation?

In some individuals, we find, instead of softening of the mucous membrane, a wasting or thinning of the coats of the stomach. In these, the muscular tunic is reduced to a few pale and scattered fibres; often indeed, in several portions of such stomachs, we find the parietes of the organ composed only of the peritoneal coat, and a slight layer of cellular substance. These phenomena are generally seen in those who have died of lingering chronic diseases. In one instance, however, our authors found this condition of the stomach in a young girl of considerable embonpoint, and who came into the hospital with symptoms of acute meningitis, of which she died. The previous history of the case was not ascertained.

"Another proof of the non-inflammatory nature of many of these softenings and thinnings of the coats of the stomach, may be drawn from the cures or alleviations of many gastric disorders, by remedies which are the reverse of antiphlogistic. We are disposed, say our authors, to regard as an affection quite different from gastritis, that ensemble of symptoms which practitioners have long designated by the term '*embarras gastrique*,' (or what we call in this country *stomach disorder*, or, indeed, *indigestion*) consisting of loss of appetite, bad taste in the mouth, loaded tongue, (the papillæ not elevated or red)—irregularity of the bowels, the stools being sometimes scanty and hard, sometimes frequent and loose—sensation of constriction or weight at the epigastrium—and occasionally nausea. To these may be added, a general malaise—habitual lassitude—sallow or yellow complexion—features expressive of distress—eyes lack lustre—pain or other affection of the head. This train of symptoms we have often seen resist the applications of leeches to the epigastrium, low diet, diluent drinks, &c. and rapidly give way to the exhibition of an emetic, or a brisk purgative. Is there not, in such cases, a vitiated saburral secretion from the mucous membrane of the stomach and bowels? Or are the vital powers (whatever they be) of digestion morbidly altered? Do vomits and purgatives, by exciting the stomach and bowels, together with the auxiliary neighbouring organs, re-establish the power of digestion? Do these remedies change, in some unknown way, the mode of secretion in the liver and pancreas? We know not. But this we know, that the treatment above-mentioned is very efficacious in these kinds of cases, and that the antiphlogistic treatment is useless, if not injurious."

*Case* 6. A young man, 22 years of age, had experienced, for three weeks before he entered La Charité, severe pain in the head, and frequent giddiness, together with loss of appetite, bad taste in his mouth, obstinate constipation. When examined in the hospital, his countenance was depressed, the tongue loaded, without any redness—abdomen soft, and free from tenderness—pulse a little accelerated—no heat of skin—great giddiness—violent pulsating pain in the head.* Leeches were applied to each side of the neck, and next day a large bleeding was taken from the arm. No amelioration ensued. During the three succeeding days, pediluvia, lavements, ptisans, &c. were employed, but all to no purpose. Seven days elapsed, and still the patient was in the

* Those who have weak stomachs, and who are unfortunate or rather imprudent enough to commit an occasional debauch, know the terrible pulsating pain of the head which they experience the next morning, and which is greatly aggravated by the least motion. It often leads practitioners to apply leeches, whereas, the best remedy is some light stimulant. It is quite different, in its nature, from fulness of blood, or inflammation, and generally goes off, after the next dinner.—*Rev.*

same condition as when he entered the hospital, although the antiphlogistic treatment was pursued, including leeches. On the tenth day, or more than a month from the commencement of the complaint, the patient was seized with spontaneous vomiting of a large quantity of green bile, which was followed by a smart purging of yellow liquid matters. Next day every symptom of his malady was gone, except a diarrhœa, which also ceased spontaneously in a few days more. The patient was discharged cured—not by the doctors, but by Dame Nature. "Would not an emetic, followed by brisk purgation, in the beginning, have cured the above disorder?" We think there is little doubt of it.

*Case* 7. A young man, twenty years of age, who had enjoyed good health, had become addicted to masterbation, and presently his digestion became deranged—he felt a load at his stomach after eating—he had great depression of spirits—and to these was added violent cephalalgia. These symptoms had continued some months, when a physician was consulted. Being now much alarmed, he discontinued his secret vice, but the cephalalgia and stomach disorder persisted, and he was supposed to be labouring under chronic gastritis. He was therefore put upon rigid diet, and leeches were repeatedly applied to the epigastrium. No success followed this treatment. The head-ache and the stomach disorder continued as bad as before. The regimen was then changed, and the patient was allowed a more substantial diet, consisting of animal food and broths. In a short time after this change was made, the head-ache disappeared—the symptoms of indigestion vanished—and the young man was restored to perfect health.*

Unquestionably the above case presents a state of stomach dependent on *debility and irritability*, rather than on any inflammatory action in the organ. This condition was kept up by the antiphlogistic treatment, and remedied by a change to light animal food, which is infinitely less irritating than the diluents and slops of the antiphlogistic regimen.

Our authors know a middle-aged man, of weakly constitution, who, for some time, had lost his appetite, and experienced much uneasiness, after taking food. The administration of sulphate of quinine restored his appetite and his health together. We have seen numerous cases of the same kind. Well may our authors ask—"can such a complaint as the above be any grade of gastritis?" The following case is calculated to produce some sensation on the Continent.

*Case* 8. The Countess of ———, aged 29 years, was the patient. Her father had died of an organic disease of the stomach. She was married at the age of 17, and had borne four children in the first five years of her marriage. She had contracted a blennorrhagia three years ago, which had been treated first with cooling diluents, and afterwards with astringent injections. The discharge ceased, and she enjoyed good

---

* See the paper on gastralgia mistaken for gastritis, in a former number of this Journal.

health for two years afterwards. She then experienced some moral afflictions of a distressing nature, from which time she began to lose her embonpoint—her complexion became sallow—and her digestion greatly disordered. She lost her appetite—food caused great uneasiness in the stomach, and was sometimes rejected by vomiting—no fulness in the epigastrium; but there was tenderness on pressure there—frequent eructations—tongue white—stools natural—pulse scarcely accelerated—skin dry and harsh—menses regular, but scanty. "Every thing seemed to announce the existence of chronic gastritis." "There were no symptoms of hepatic disease." Leeches were frequently applied to the epigastrium, and they seemed sometimes to diminish a little the morbid sensibility of the stomach and bowels. Tartar emetic plasters, blisters, counter-irritation of every kind, were employed, as well as sedatives internally, but to no effect, and the disease made progress. In the course of four months from the commencement of the symptoms, the vomitings became quotidian, and almost every species of food was rejected. Asses' milk was the only thing that could be retained.

The physicians were despairing of a cure, when, one day, the patient complained of a burning sensation in her throat, and a difficulty of swallowing. On inspection, a roundish ulceration was discovered in the posterior part of the pharynx, whose aspect had a syphilitic appearance. A question now was raised whether the disorder of the stomach might not be of a venereal nature. The physician clung to this idea, as affording the only hope of a cure. Mercury, in very small doses, was therefore exhibited, and continued for six weeks, during the first two or three of which, there was no aggravation or mitigation of the stomach disorder; but about the 27th day of the new treatment, the vomitings became less frequent and severe—the stomach began to shew some power of digestion—the muscular force improved—the complexion became more clear. By the 40th day, the improvement, in all respects, was most unequivocal. Frictions were then added to the internal use of mercury, and after the 11th or 12th inunction, the vomitings entirely ceased—aliments could be retained and digested—the epigastric region became soft and void of tenderness—and, in short, the Countess was restored to perfect health.

We shall not stop to enquire whether the malady of the patient was syphilitic or hepatic; the probability being greatly in favour of the latter supposition. That the cure was owing to the mercury there can be no doubt.

### DISEASES PRODUCED BY LEAD.

The divers preparations of this metal, our authors observe, which are introduced into the animal economy, in a state of extreme division, produce, both in the digestive organs and other parts of the body, a host of anomalous and strange affections, the cause of which has been too confidently attributed to chronic inflammation of the intestines. To this pathology of the diseases produced by lead, the attention of physicians has been almost exclusively directed; but our authors think that the facts which they are enabled to bring forward, on this occasion, may perhaps shake the foundation of this exclusive pathology.

COLICA PICTONUM.

The history of this disease has been traced with care, by many writers; but still the following questions may be asked:—What is the nature of the disease? What is the exact condition of the digestive tube, in those who fall victims to the malady? What sort of lesions are those which are *consecutive*, and especially of the nervous system? Are these disorders of the nervous system *always* consecutive of the disease in the digestive organs? Are they not sometimes primitive? What is the best mode of treatment? Are the means which remove the colica pictonum adapted also to remove the consecutive or primitive affection of the nervous system? Is the colic produced by copper, in which disease there is diarrhœa, instead of constipation, to be healed by the same remedies as the colica pictonum? Finally, do not the symptoms and the treatment of the class of diseases under consideration, and the state of the intestines after death, throw some light on the nature of those various abdominal pains which cannot be accounted for as the results of either peritonitis or enteritis?

1. POST-MORTEM APPEARANCES.

We are told that, in those who die of colica pictonum, the intestines are found contracted in their calibre. Traces of inflammation, indeed, have been found in some cases, but not in others—hence we may conclude that the phlogosis is only an accidental complication, and not necessarily connected with the disease. Of more than 500 individuals who have been affected with colica pictonum, during the last eight years in LA CHARITÉ, five only have died, while under treatment—and two of these did not die of the colica pictonum, but of complaints quite independent of that disease.

*Case* 1. A painter, aged 33 years, had been already twice at LA CHARITÉ for the treatment of colica pictonum. He came in for the third time, in the year 1820, presenting all the usual symptoms of the disease, as violent abdominal pains, not augmented by pressure—shrunk state of the abdomen—vomitings—obstinate constipation—tongue natural—pains in the limbs—apyrexia. He had no motion during the last fortnight, and the abdominal pains were of five days' standing. He had taken castor oil, without effect. Immediately after his entrance, he was put on the usual treatment of LA CHARITÉ. On the third day, he complained of sudden and unusual pain in the region of the heart, and in a few minutes afterwards he expired.

*Dissection.* It was found that the root of the aorta, within the pericardium, had given way, and that bag had been filled with blood.

A most careful examination was then made of the parts concerned in the original malady, as this was the first time they had had an opportunity of examining a patient who had died *during* the treatment of colica pictonum.

They were rather astonished to find a total absence of all contraction or straitening of the intestinal canal. The knuckles of great and small intestines were, on the contrary, more dilated than usual. The peritoneum was healthy. The internal surface of the stomach was pale, and of natural consistence and thickness. It was covered with ropy mucus. The duodenum was sound in all respects. In a few points of the small intestines, there were faint and very trifling arborisations of the vessels under the mucous membrane. The large intestines contained but a small portion of hard fæcal matters.

Now this patient died when the colica pictonum existed in a very intense degree, and yet there could scarcely be said to be a blush of redness on the mucous membrane of the intestines—and that too, after the patient had been taking drastic purgatives. It cannot be said, in this case, that the mucous membrane had been blanched by the hæmorrhage of which the patient died; for there were not eight ounces of blood in the pericardium.

*Case* 2. A man, of middle age, who worked in a white lead manufactory, had experienced, for some days, acute abdominal pains, and all the symptoms of colica pictonum. The disease was so well marked that the house-pupil noted it on the man's ticket the day he came into the hospital. The very next day this patient was seized with apoplexy, and died in forty-eight hours afterwards. Purgatives and glysters, in this interval, had procured no stools.

*Dissection.* A large extravasation of blood was found in one of the hemispheres of the brain. The internal surface of the stomach presented a slight degree of injection, towards the great CUL DE SAC. In other respects, this membrane was in its natural condition throughout. The mucous membrane of the small intestines presented some slight traces of injection, particularly in the venous system. The membrane was otherwise pale throughout. The same was the case with the large intestines. There was no contraction of calibre in any part of the intestinal canal. As far as these two cases go, they certainly afford no ground for considering colica pictonum as dependent on enteritis.

*Case* 3. A man, 50 years of age, a plumber by trade, entered LA CHARITÉ, for violent colicky pains, under which he had laboured for some days. The common treatment of the hospital was commenced. On the third day he was relieved a little, but still suffering severely. In this state he was taken suddenly with nervous symptoms of a grave character, which carried him off in two hours.

*On dissection*, the mucous membrane of the stomach presented nothing particular. In the intestines, a few isolated points were injected red, but not to such an extent as to shew the colour through the peritoneal coat. The rest of the tube was transparent. In the transverse arch of the colon, there were a few inches of the inner membrane red. No other appreciable alteration could be found in any of the three cavities.

In this case it will hardly be contended that the *post mortem* appearances could at all account for the symptoms of colica pictonum during life.

*Case* 4. A ship-painter, aged 38 years, entered LA CHARITÉ, for the treatment of colica pictonum, the symptoms of which were very well marked. On the succeeding day he had an attack of epilepsy. For some days afterwards the abdominal pains continued their course, but were not very severe. The usual hospital means were employed. Ten days after he entered the hospital, the colica pictonum still in existence, the man fell down suddenly and died.

*On dissection*, the mucous membrane of the stomach, near the pylorus, was of a slate colour, in a space double the size of a crown piece. The small intestines were pale, with the exception of a few spots of injection, and a few of the valves of Peyer, with minute black specks in their centre, near the valve of the colon. The internal surface of the transverse colon presented a small patch of red, and there the mucous membrane was softened. It is needless to observe, that more strongly marked appearances of inflammation than the above are often seen, where no colica pictonum, or any symptom of enteritis existed during life.

*Case* 5. A plumber, aged 50 years, had had attacks of colica pictonum several times, and was now in the third week of an attack, when he entered LA CHARITÉ. The pains were not severe, but they were constant, and exasperated from time to time, so as to cause the patient to cry out. The constipation was obstinate, and the upper extremities were paralyzed, as to muscular power. The ordinary treatment of the hospital was commenced. On the 4th day, he was seized with symptoms of asphyxia, and died suddenly.

The stomach was found distended with fluids. Towards the great *cul de sac*, there was a portion of the mucous membrane softened, though white, except two small round spots. All the rest of the gastric mucous membrane was of natural appearance. The intestines, small and large, were dilated rather than contracted. In the mucous membrane of the small intestines, there were some injected patches. The rest of the membrane, throughout the whole canal, was unaltered.

Thus, in the whole of these five cases, there were evidently no *post-mortem* changes to account for the symptoms of colica pictonum. To these five, our authors add one, observed by M. Louis. The patient was in the eighth day of an attack of colica pictonum, when he died suddenly. No disease was found in the intestinal canal.

### Symptoms.

Our authors do not enter into an analysis of the common symptoms of this disease, which are so well known; but confine themselves to remarks on some particular phenomena.

They observe that it is not correct to say that the abdominal pain, in colica pictonum, is always diminished by pressure—in some, pressure *increased* the pain. Yet, in all these cases, the other phenomena of the disease were similar. Neither is it true that the abdomen is always shrunk in the malady under review. In many cases, the abdomen is unaltered in volume: in some, it is distended beyond the natural size. The most constant and unvarying symptom was the constipation. It precedes the abdominal pains—and these last diminish in proportion as the constipation is overcome. But the digestive tube is not the only organ which suffers in colica pictonum. The nervous system is often affected in a remarkable manner; and thus a great variety of phenomena are produced, according to the idiosyncrasy of the individual and other circumstances, in the locomotive as well as in the nutritive system. The most common of these nervous disorders is the pain in the limbs, and especially in the arms. These pains often precede the colic: sometimes they constitute the whole of the apparent morbid phenomena, the digestive organs being unaffected. Hence our authors rationally conclude that the disorders of the nervous system are not purely sympathetic of the disorder in the digestive tube. These pains are often accompanied by a remarkable debility of the muscles in the vicinity of which they are situated—which debility gradually changes into veritable paralysis. Thus we find the *sensibility* of certain members exalted, while their muscular powers are diminished or annihilated. What takes place in the limbs bears no small analogy to what obtains in the bowels, where we find great *pain*, accompanied by want of contractile power in the muscular coats of the intestines—at the same time that the mucous membrane is much less sensible to the presence of even drastic purgatives.

The most ordinary site of paralysis, in those who handle lead, is in the extensor muscles of the hand—hence results, from loss of balance of power, an habitual contraction of the flexor muscles, the hand being clenched, and the wrist bent on the fore-arm. This paralysis does not generally occur till after the individual has been a long time subjected to the emanations and contact of lead, and till he has had repeated attacks of the colica. Nevertheless, our authors have seen some exceptions to this statement. The paralysis is generally very slow of cure. It is not always confined to the wrists—it sometimes invades the whole of the upper extremities, and causes total immobility of those members. The sensibility of the skin is generally preserved, as was before observed. The intellects are hardly ever affected.

After what has been said of the symptoms, it is rather in the spinal marrow than in the brain that we can expect to find any traces of organic change after death. The encephalon, examined with the greatest care, presented no lesion to our authors' view. The spinal canal contained a small quantity of limpid serum, such as is generally found in all bodies. The envelopes of the spinal marrow were pale—and the medulla itself was completely free from every physical lesion that could be detected by the eye. Neither could any thing be discovered in the nervous plexuses of the neck—the nerves emanating from the spine—or the pneumo-gastric nerves. With the exception of those appearances which have been already described in the stomach and bowels, there was no appreciable mark of disease in any organ of the body. "No doubt," say our authors, "there was serious alteration in the cerebro-spinal axis; but this alteration could only be demonstrated by the symptoms, and not by dissection." In a few instances, our authors saw complete or incomplete paraplegia in colica pictonum, with exaltation of the sensibility, and great pain in the members paralyzed.

Instead of paralysis, they have seen some instances of convulsive movements, and epileptiform attacks, in those who had been subjected to the poison of lead. The following is an example with the *post mortem* examination.

*Case.* A ship-painter, aged 38 years, had colica pictonum when he entered LA CHARITÉ. The following day, he was seized with epilepsy, the attack lasting a long time, and being succeeded by apoplectic symptoms, which continued from 30 to 40 hours. In this attack the patient appeared several times to be in articulo mortis; yet he recovered his senses and muscular power, the colicky pains continuing, though not in a violent degree. Some days passed thus, when, one evening, just as he was getting into bed, he fell down, and instantly expired.

The body was opened 14 hours after death. The meninges of the brain were pale—the encephalon presented no sign of congestion—very little serosity in the ventricles. The rest of the brain, the spinal marrow, and the nerves emanating from them were carefully examined, but no lesion could be detected. The thoracic and abdominal organs were all sound, with the exception of some traces of injection in the mucous membrane of the intestines, as before recorded. The foregoing, as also the following case, are among those already mentioned, as having died suddenly. The dissection then only related to the state of the digestive tube. They are here introduced, to shew the investigation of the cause of sudden death.

*Case.* A plumber, having the colica pictonum, when he entered LA CHARITÉ, was seized, three days afterwards, with complete insensibility, and loss of all muscular power, of which he died in one hour. The brain and spinal marrow were examined with the greatest care, but no appreciable alteration from a state of perfect integrity, could be detected. M. Louis has made similar observations.

In a few cases, admitted into LA CHARITÉ, of colica pictonum, the symptoms were rather different from those already enumerated. There were palpitations, violent cephalalgia, dyspnœa, returning in paroxysms—cough of a nervous character, resembling that which sometimes attends globus hystericus—a numb sensation about the heart, resembling that in the arms, and somewhat analogous to the sensations experienced in an attack of angina pectoris.

### Treatment of Colica Pictonum.

Several physicians now follow the treatment of De Haen—namely, the purely antiphlogistic method; and it is indisputable that many patients recover perfectly under this system. But then it is to be remarked that, when the colica pictonum is in a moderate degree, the patient will get well in an hospital or in any other situation, where he is no longer exposed to the poison of lead, and that without any medical treatment whatever. This being premised, our authors do not set themselves up as censors of this or that method of treatment. They present themselves merely as historians of what they have actually seen. They are not constructing systems of pathology; but only collecting materials for such systems. Nevertheless, our authors think they are justified in drawing the following conclusions from the facts which have presented themselves to their notice in La Charité.

1mo, Colica pictonum, treated by sanguineous depletion (leeches) and emollient drinks, is much more tedious of cure than when treated on the plan of La Charité.*

2ndo. Many cases which have entirely resisted the antiphlogistic method, cede to that of La Charité.

3tio. We have never seen this latter method fail. Sometimes indeed it is necessary to go through the process two or three times, in order to effect a complete cure. In many cases, however, the symptoms disappear as soon as evacuations are procured upwards and downwards.

4to. Conducted with prudence, and administered opportunely, the Charité plan of treatment never produces any bad consequences. The drastic purgatives which are given never inflame the intestinal canal, kindle up fever, or load the tongue.

Men frequently come into La Charité who have had numerous leeches applied to the abdomen, aided by warm baths, and milk diet. Some of these are relieved; but none completely cured. The constipation persists, and the abdominal pains are still complained of by

* This treatment, which indeed goes by the name of La Charité, is rather complicated; but consists almost entirely of emetics, cathartics, diluents, and anodynes. Thus, on the patient's first reception, a purgative enema is given, which consists of infusion of senna, sulphate of soda, and tartar emetic. In the course of the same day, the patient takes two pints of a diluent laxative ptisan, in which there is an ounce of sulphate of magnesia, with three grains of tartar-emetic. In the evening, he has an anodyne glyster; and by the mouth some theriaca andromach. and a grain and a half of opium. Next morning, an emetic is given, after the operation of which, he has a sudorific ptisan, through the day. In the evening the opiate and enema as before. This process goes on, with little variation, for six or seven days. One, two, or three courses of this treatment are generally sufficient for the cure.—*Ed.*

these patients. Once entered in LA CHARITÉ, and put upon the treatment by drastic purgatives, &c. they soon get entirely cured.

We shall pass over some cases which our authors relate, in illustration of the effects of strychnine, brucine, &c. as no very favourable results appear from these heroic remedies.

Finally, our authors come to the conclusion, that colica pictonum is not an entero-gastritis, but a disorder of the nervous system which presides over the functions of the digestive organs. The obstinate constipation appears to them to depend on the annihilation of muscular power in the intestinal coats, or suspension of the mucous secretion of the bowels—or, perhaps, on both.

*On some morbid states which, from their symptoms and treatment, have more or less analogy to the painter's colick.*

Some of the symptoms of colica pictonum occasionally appear (our authors remark) in people who have *not* been subjected to the emanations or contact of lead. Hence they conclude that the disease may possibly arise spontaneously. The following is given as an example, and on it we shall take the liberty of making some remarks.

*Case.* A nail-smith, aged 38 years, of strong constitution, had had no passage through his bowels, for some days; and was seized, on the 22d of June, with severe abdominal pains, particularly about the umbilicus. He took an opiate, and the pains diminished a little; but in the night, they returned with increased violence. Our authors saw him the succeeding morning, 23d June. His face was pale, and expressive of distress—eyes dull, and sunk—abdominal pains insupportably severe—no increase or diminution by pressure—obstinate constipation—tongue natural—apyrexia complete. Two ounces of castor oil were prescribed, and several alvine evacuations were procured. He was greatly relieved, and slept well in the night. From this time the bowels were kept open, and he had no return of the pain.

We are rather surprised that M. Andral should bring forward the above case, as one which bears an analogy to colica pictonum. We consider it as one of simple spasmodic colic, from retention of matters in the colon, producing irritation and spasm there. A continuance of the disease would have led to inflammation, which is contrary to the course of the painter's colic. There was also an absence of all symptoms of paralysis.*

---

* But whether the above disease bore any analogy to colica pictonum or not, it is evident that it was not produced by lead. And here we shall venture to record it as our opinion! that the case reported from the Royal Infirmary of Edinburgh, by SCOTUS, was not one of colica pictonum at all. We shall give the case, as in the original report, in No. 179, of the LANCET.

*Case. Eliz. Campbell*—" She was admitted on the 2d of November for an extensive burn of the abdomen, and contiguous parts of the body. The

It is true, our authors relate cases where the constipation continued most obstinate at intervals, for a month or more, ultimately giving way to purgatives. Still, we cannot look upon these as having any real affinity to colica pictonum. Our authors mention a phenomenon which they have observed in some of these cases, and which is worth adverting to in this place. It was this:—when the pains came on periodically, a tumour, apparently formed by a distended knuckle of intestine, would shew itself in some part of the abdomen, and persist as long as the pain was felt, disappearing as soon as the pain ceased. In such cases, there was no symptom whatever of fever. Now, we think that this very phenomenon shews that these cases, bearing, as is said, some analogy to colica pictonum, are merely instances of spasmodic colick.

We believe there are many *tumours* of this kind, which are said to be organized morbid growths, and which suddenly appear and suddenly disappear, under certain modes of treatment that gain great credit, with little merit, on such occasions.

"There is another species of colick, which presents symptoms evidently inflammatory—and recognizes for cause the handling of copper.

---

ulcer, in the usual time, assumed a healthy aspect, and continued to cicatrize in the usual manner, and nothing particular occurred in her case so far, except that her convalescence seemed more tedious than might be expected, even from so serious an injury, having never regained her wonted energy. Her pulse continued quick and feeble; her tongue slightly furred; her colour of a cadaverous hue; her features sharp, anxious, and attenuated; and, on asking her several times as to the cause of these appearances, she could assign no other reason for their presence than weakness and long confinement to bed. All her symptoms became more intense, and she now began to complain of a tendency to *costiveness, and of weakness in the extremities, from her knees downwards. On the 16th of January, the abdomen became tender and tympanitic, and she complained of violent pain.* An enema in the night brought away a large dejection, but she remained much in the same state, and was ordered a colocynth pill every sixth hour.—17th. The pain in the abdomen remained unabated; the bowels not opened during the night, and passed but about four ounces of urine. An injection of sulphate of soda and senna, and a dose of castor oil, were ordered to be taken immediately. From this to the 19th, she had taken at intervals castor oil, calomel, tincture of aloes, and had fomentations, and several enemata of turpentine administered, but without any effect. At eight o'clock on the night of the 20th, she was visited by Dr. Hunter, and ordered eight grains of scammony, and three of hyoscyamus every third hour, but, becoming worse during the night, an infusion of thirteen grains of tobacco was given in the form of injection, which freely evacuated the bowels in the course of about two hours. Since that period she remains in a state of extreme debility, *and with a tendency to constipation.*"

In a few days after this report, she died. Now we appeal to the practical reader, after what has been stated in this article by M. Andral, whether the above case bears any analogy whatever to the disease called colica pictonum? —The description requires only to be set forth, to carry conviction in the negative. It is true that Dr. Ballingall, in his clinical reports, alludes to this case as one of colica pictonum; but, with all deference for his authority, we think he was quite mistaken.

It is very often met with in La Charité, among artisans who work with this metal. It differs from colica pictonum, 1mo, by the lesser intensity of the pains—2ndo, by the existence of diarrhœa—3tio, by the accompaniment of febrile symptoms—in short, by its being manifestly the result of a *veritable* inflammation of the intestines. Yet, even in this disease, we have seen M. Lerminier give, with success, the most energetic purgatives, which form the basis of the treatment for the saturnine colick." 509.

We shall introduce a single case in illustration.

A copper-founder, aged 50 years, had enjoyed habitual good health. For about a fortnight before he entered the hospital, he had been afflicted with abdominal pains, which occasionally became so severe as to cause a tendency to fainting. For ten days, he had tenesmus of a very distressing nature. He was tormented by a constant desire to go to stool, nothing being passed but mucus streaked with blood. The abdominal pain was not increased by pressure—tongue clean—features shrunk—slight acceleration of pulse, with but little increase of heat. On the first day, M. Lerminier applied fifteen leeches to the anus. Next day there was no amendment. He then determined to put in force the treatment for saturnine colick. On the third day, after abundant evacuations had been procured, upwards and downwards, the abdominal pain became much lessened, and the tenesmus disappeared. The treatment was continued, and the dysenteric symptoms soon ceased. The patient had a speedy recovery.

We had hopes of being able to include in this article the important subject of peritoneal inflammation, acute and chronic, which occupies nearly 200 pages of the present volume; but find ourselves obliged to defer it to a separate article in our next number. There is a great mistake every day committed, by including in the term Enteritis, the phlogosis of the peritoneal and mucous membranes of the intestines. Yet the diseases are *very frequently* quite independent of each other—and are always very different in their symptoms, in their termination, and in their treatment. We shall therefore make no apology for dedicating an entire article in our next number to the subject of peritoneal inflammation, than which there is not a more dangerous phlogosis incident to the human frame, or one which requires greater vigilance or discrimination on the part of the practitioner.

